# Determining a risk-proportionate approach to the validation of statistical programming for clinical trials

**DOI:** 10.1177/17407745231204036

**Published:** 2023-11-13

**Authors:** Carrol Gamble, Steff Lewis, Deborah Stocken, Edmund Juszczak, Mike Bradburn, Caroline Doré, Sharon Kean

**Affiliations:** 1 Liverpool Clinical Trials Centre, University of Liverpool, Liverpool, UK; 2 Edinburgh Clinical Trials Unit, University of Edinburgh, Edinburgh, UK; 3 Clinical Trials Research Unit, University of Leeds, Leeds, UK; 4 Nottingham Clinical Trials Unit, University of Nottingham, Nottingham, UK; 5 Clinical Trials Research Unit, ScHARR, University of Sheffield, Sheffield, UK; 6 Comprehensive Clinical Trials Unit, University College London, London, UK; 7 Robertson Centre for Biostatistics, University of Glasgow, Glasgow, UK

**Keywords:** Risk-based approach, statistical programming, clinical trials, validation

## Abstract

**Background:**

The contribution of the statistician to the design and analysis of a clinical trial is acknowledged as essential. Ability to reconstruct the statistical contribution to a trial requires rigorous and transparent documentation as evidenced by the reproducibility of results. The process of validating statistical programmes is a key requirement. While guidance relating to software development and life cycle methodologies details steps for validation by information systems developers, there is no guidance applicable to programmes written by statisticians. We aimed to develop a risk-based approach to the validation of statistical programming that would support scientific integrity and efficient resource use within clinical trials units.

**Methods:**

The project was embedded within the Information Systems Operational Group and the Statistics Operational Group of the UK Clinical Research Collaboration Registered Clinical Trials Unit network. Members were asked to share materials relevant to validation of statistical programming. A review of the published literature, regulatory guidance and knowledge of relevant working groups was undertaken. Surveys targeting the Information Systems Operational Group and Statistics Operational Group were developed to determine current practices across the Registered Clinical Trials Unit network. A risk-based approach was drafted and used as a basis for a workshop with representation from statisticians, information systems developers and quality assurance managers (n = 15). The approach was subsequently modified and presented at a second, larger scale workshop (n = 47) to gain a wider perspective, with discussion of content and implications for delivery. The approach was revised based on the discussions and suggestions made. The workshop was attended by a member of the Medicines for Healthcare products Regulatory Agency Inspectorate who also provided comments on the revised draft.

**Results:**

Types of statistical programming were identified and categorised into six areas: generation of randomisation lists; programmes to explore/understand the data; data cleaning, including complex checks; derivations including data transformations; data monitoring; or interim and final analysis. The risk-based approach considers each category of statistical programme against the impact of an error and its likelihood, whether the programming can be fully prespecified, the need for repeated use and the need for reproducibility. Approaches to the validation of programming within each category are proposed.

**Conclusion:**

We have developed a risk-based approach to the validation of statistical programming. It endeavours to facilitate the implementation of targeted quality assurance measures while making efficient use of limited resources.

## Introduction

The contribution of the statistician to the design and analysis of a clinical trial is acknowledged as essential. Ability to reconstruct the statistical contribution to a trial requires rigorous and transparent documentation as evidenced by the reproducibility of results. Statistical activities including data transformation, analysis and reporting and corresponding quality control activities form key components of the clinical trial data life cycle and fall within the scope of a Good Clinical Practice inspection by regulatory authorities.^1–3^ The objective of examining statistics functions during Good Clinical Practice systems inspections is to establish that the processes in place give assurance that the trial data are managed and analysed to give accurate and credible trial results.^3^ This is an important step in the reproducibility of research.^4^

Challenges relevant to data integrity faced by academic clinical trials units (CTUs) for compliant data management and underlying information technology and systems infrastructure have been previously reported^5,6^ and guidance developed.^7^ However, data integrity challenges remain for clinical trials globally and these issues continue to be a source of regulatory focus and compliance findings. New guidance focusing on data quality and integrity^6,8–11^ has been published by regulatory bodies with a common thread being an emphasis on a risk-based approach.^12–16^

While there has been a focus on compliant systems and data management, there has been limited attention regarding statistical processes specifically. In 2014, a member of the Good Clinical Practice Medicines and Healthcare products for Regulatory Agency (MHRA) inspectorate was invited to talk to the United Kingdom Clinical Research Collaboration (UK CRC) Registered Clinical Trials Unit (RCTU) Network about regulatory inspections and findings of particular relevance to statisticians within academic RCTUs. The talk highlighted not only the data integrity strengths but also the potential to further improve practice as control of the data moved from data management to statisticians, and in particular approaches to validation of statistical programming. This raised concerns regarding the extent of validation requirements and resources necessary to achieve compliance within the academic setting. There is little content within Good Clinical Practice that relates to the practice of statisticians^17^ and in particular translates into statistical programming and their validation responsibilities directly.

Statistical programmes can be considered as ‘one-off’ programmes, defined as a programme used with a specific set of data from a single study.^18^ As a generic definition, any syntax used to deliver an output is considered to be programming, and every programme has the potential for human error. The likelihood of and impact of error vary considerably, depending on the method of programming and the intended use of the programme.

While some form of validation is important, formal methodologies used for software development, for example, waterfall,^19^ agile,^20^ and in particular their testing approaches may be excessive for the purposes of statistical programming. However, there is a need to acknowledge that cross-disciplinary knowledge exchange is useful and can be referenced in order to improve robustness of statistical programming practices and provide resources^21^ to support their validation.^22,23^

A risk-proportionate approach is acceptable to regulators^12,13,14,15,16^ Regulatory guidance states that ‘it is acceptable for quality control checks to be undertaken using a risk-based approach with the detail and level of checking varying depending on the item being checked’.^5^ Our objective was to develop a framework to achieve a risk-proportionate approach to the documentation and validation of statistical programming consistent with regulatory requirements but realistic in the confines of resources allocated to this type of activity within academic CTUs. These units have a breadth of trial experience, differing levels of programming expertise and are not comparable in terms of staffing and available resource. Our intention was to develop a framework which did not dictate a particular algorithm to inform which approach was best but would provide a pathway as to what would be acceptable to ensure trial results were reproducible.

## Methods

The delivery of this project was centred on the engagement and collaboration of two operational groups of the UK CRC RCTU network, the Statistics Operational Group and the Information Systems Operational Group. The aim of the operational themed groups is to share best practice among its RCTU membership (48 UK CRC RCTUs at the time of this work) and to recommend standard approaches to common issues.

In April 2015, the Chair of the Information Systems Operational Group attended a statisticians’ network meeting to discuss statistical programming. Attendees interested in contributing to the project were asked to share relevant MHRA experience as well as materials, including related inspection findings, standard operating procedures, statistical programming documents developed in-house or otherwise that they felt were relevant, and relevant publications. In addition, a review of the published literature, regulatory guidance and knowledge of relevant working groups was undertaken.^24–26^

Two surveys were developed to determine current practices across the RCTU network. One survey targeted clinical trials’ statisticians and the other targeted information systems’ managers. Both surveys were piloted within their respective operational subgroups before being circulated for completion by attendees at the network meetings taking place in October 2017. The survey results were presented, followed by round table discussions to allow attendees to elaborate on responses and offer additional information on their practices.

The information from these meetings was then used to draft the risk-based approach which formed the basis of a small workshop (April 2018). Attendees for this Workshop 1 (n = 15) were selected to ensure representation from statisticians (n = 7), information systems (n = 5), quality assurance managers (n = 2) and an RCTU secretariat representative. Consideration was also given to ensuring variety in RCTU selection (n = 7) in terms of their trial portfolios and trials experience.

Following discussion at this workshop, the approach was modified. A second, larger scale workshop (n = 47) was held in June 2018, aiming to achieve a wider perspective and discussion of the approach and its implications for delivery. Workshop 2 supported attendance from a larger number of RCTUs (n = 35) and importantly engaged with the MHRA inspectorate to create dialogue around the issues identified. Attendees at this meeting also included the RCTU Quality Assurance subgroup Chair, the Chair of the Data Sharing group, a member of the RCTU network secretariat and an IS consultant leading a National Institute for Health and Care Research project across the RCTU network. The approach was revised based on these discussions.

## Results

### Survey findings

The results of the RCTU surveys (n = 31 of 48 RCTUs responded) indicated variation in the approach and extent of validation practices. Programmes for final analyses were routinely validated (97%), with routine validation of programmes for interim analyses reported less frequently (57%).

Free-text responses indicated 77% (24/31) used independent programming, with 33% (10/31) using code review and 7% (2/31) having existing code rerun by another statistician. Where independent programming was used, the majority focused on the primary outcome and only 13% (3/24) indicated that the approach or the extent of its application was considered within a formal risk assessment.

### Identification of relevant resources

The following resources were identified through the literature search and/or suggested by the RCTU operational groups:

- Good Clinical Practice Guide, MHRA, TSO information and publishing solutions, 2012, known as the ‘grey book’.^3^
- Computerised Systems validation in clinical research, A practical guide, 2nd edition, Association for Clinical Data Management.^18^
- Guideline for good clinical practice E6 (R2) (EMA/CHMP/ICH/135/1995).^1^
- Statistical Principles for Clinical Trials E9 (CPMP/ICH/363/96).^2^
- The Global Healthcare Data Science Community, Pharmaceutical users software exchange.^26^

While there are no recommendations or regulations specific to validation of statistical programmes, it was established that statistical programmes do fall under the definition of a one-off programme: a programme to be used with a specific set of data from a single study.^18^ With a one-off programme, the approach to validation corresponds to the programme’s complexity and the consequences of an error. Examples of a range of MHRA-acceptable methods to validate statistical programming are summarised in Table 1.

**Table 1.** Approaches to validation of statistical programming (Page 327 Good Clinical Practice Guide, MHRA, TSO information and publishing solutions, 2012, known as the ‘grey book’^3^).

1. Independent programming (dual programming and comparing output)
2. Detailed checks of output against raw data or data listings combined with review of code/programming (essentially computer system validation). It is expected that the programmers writing code will undertake checks to confirm that the programme is functioning correctly.
3. Use of previously validated code or macros
4. Checks and retention of any logs produced by the statistical software to ensure the code runs correctly
5. Checks on accuracy and quality of any new variables/data sets derived from final data sets provided at the end of the data management process
6. Review of any formulae in spreadsheets (where used for analysis)

### Development of a risk-proportionate approach

A risk-proportionate approach typically involves: (1) identification of the risk or error; (2) assessment of the likelihood of the error occurring; (3) consideration of the impact of such errors on trial participants and trial integrity and (4) judgement of the extent to which such errors would be detectable. In applying these steps to statistical programming, we first identified categories of statistical programmes defined by their purpose (see Table 2).

**Table 2.** Categories of statistical programming and potential errors.

Programme category	Description of programme purpose	Potential failures/errors
Randomisation	Programmes to generate the randomisation schedule.	Failure to randomly allocate participants (and/or to the correct allocation ratio).
Empirical/probing	Programmes written to develop the statistician’s understanding of the data and how the data collection forms are completed. They are restricted to descriptive statistics, plots, listings, etc. They may be used to explore or understand nuances in the data but do not contain comparative statistical analyses, for example, comparisons of treatment groups. Restricted to descriptive statistics, plots, listings, etc. They do not directly contribute to output and are less likely to be used again.	Failure to understand the data structures and distributions.
Complex checks	Programmes written to examine/clean the data as part of data validation activities to support data management.	Failure to identify data errors or inconsistency in the data leading to an insufficiently clean data set for analysis.
Derived data sets	Programmes written to identify membership of the analysis sets. Programmes written to calculate/transform/derive outcome measures, for example, to calculate overall quality of life scores from individual questions, time to event, etc.	Failure to correctly include/exclude participants within analysis population(s); incorrect calculations, derivations or algorithms.
Statistical monitoring	Programming used to produce reports for internal use only, Trial Steering Committee and Data Monitoring Committee reports, Drug Safety Update Reports.	Failure to identify protocol deviations/violations and signals in the data. Failure to quickly identify safety concerns.
Interim and final analysis	Programming to produce the results of analyses prespecified in the Statistical Analysis Plan.	Failure to ensure interpretation and future clinical decisions are based on correct analysis.

The categories were discussed at Workshop 1 and taken forward unchanged to Workshop 2. The programming categories were used to identify potential failures or errors and a set of questions were developed to be considered during discussions around likelihood (see Table 3). The questions were discussed in both workshops and applied to each programming category as defined in Table 2.

**Table 3.** Validation considerations.

1. Do the programmes need to be flexible and fluid? For example, is it likely that emerging issues, and additional checks may need to be added as the trial progresses? Is there a high risk that the programmes will need to evolve with the trial, for example, in response to changes to the database or protocol amendments?
2. What is the frequency with which the programme’s purpose needs to be repeated? For example, complex checks and recruitment monitoring may need to be undertaken monthly while other programmes may only be required annually or only planned for a single time point.
3. Is the content fixed and predetermined? Most statistical programmes are predetermined in response to the risk assessment, monitoring and statistical analysis plan or in discussion with the Data Monitoring Committee (DMC). However, during the life cycle of the trial, unplanned analyses may be required or requested in response to those previously undertaken.
4. How likely is there to be a need to replicate programme output? Some programmes may be at higher risk of a replication requirement during an audit. For example, programmes used to generate DMC reports or final analyses.
5. What is the complexity of the programming and data management steps involved? Where determination of an event requires derivation from multiple variables across repeated time points, the programming required will have additional complexity requiring additional time. For example, simple derivations may be low risk for error, but their simplicity means that validation is quickly achieved.

The questions originally included ‘What is the impact of an error?’, but this was removed and incorporated into the main body of the risk assessment. An additional question was added to the ‘randomisation’ category only: ‘Is bespoke programming required to generate the allocation or the allocation delivery system?’ This was considered particularly relevant to this category as validated software functions may often be used, for example to generate allocation lists, which carry very different risks to dynamic allocation methods. Table 1 provides MHRA-acceptable approaches to validation of statistical programming. Whichever validation approach is taken, it is important to consider where potential errors may occur and ensure mitigation as appropriate.

Table 5 provides the risk assessment with illustrative responses. To guide validation requirements in a risk-proportionate manner for each category of statistical programming identified in Table 1, the validation considerations described in Table 3 were applied to the risk assessment framework (impact; likelihood; detectability).

Additional points discussed at Workshop 2 included identification of the start and end points of the validation activities. For example, does this start from extracting raw data and importing it into the analysis package, from determining membership of the analysis populations or from a later point? The starting point will depend on the use of previously validated programmes while the end point of validation activities may depend on the approach used to populate the clinical study report (Table 4).

**Table 4.** Risk-based approach to the validation of the clinical study report.

Approach	Risk	Mitigation	Comments
Results taken from statistical package output and entered manually into a word document	Transcription errors	Check the programme output and logs to ensure there are no errors in the code. Double check the output reflects the results presented in the analysis report(s).	Review of programme output and logs may be unnecessary if a previously validated programme used. Statistical programmes can generate vast amounts of output, much of which may not be required. This increases potential for transcribing incorrect values into the report.
Tables are output from statistical package into a spreadsheet ready format, for example, excel or csv so that the table as a whole requires copy and pasting	Copy and paste errors	Check the programme output and logs to ensure there are no errors in the code. Double check the output reflects the results presented in the analysis report(s).	Review of programme output and logs may be unnecessary if a previously validated programme used. Comparison of output and the results is far more efficient when complete tables are produced as output.
Tables generated directly by statistical programming	No copy and paste or transcription errors. Programming errors.	Check the programme output and logs to ensure there are no errors in the code.	Review of programme output and logs may be unnecessary if a previously validated programme used.

Generating the clinical study report directly from the statistical programming was considered by attendees as best practice, given it removes the possibility of introducing transcription and typographical errors and simplifies the ability to reconstruct the analysis. The need for a risk-proportionate approach to quality control the report was discussed, and for each approach, the risks and mitigations were identified. In reality, the approach used will require consideration of the type of report, resources, amenability to change or reformatting and the requirement for repeated use. For example, the time taken to programme tables produced directly into the report may be greater than the alternatives, and less amenable to changes if required. This is likely to be led by resources available; however, these aspects could be formalised within the risk assessment framework of Table 5.

**Table 5.** Risk-proportionate approach.^a^

Randomisation		
	Impact area:	Safety Validity
	Impact^b^	Catastrophic – trial validity impacted by errors
	Likelihood^c^	Unlikely: reuse of previously validated programmes or standard commands within statistical packages
	*Considerations:*	
	a. Is bespoke programming required to generate the allocation list or the allocation delivery system?	Dependent on trial requirements.
	1.Do the programmes need to be flexible and fluid? (Is there a high risk that the programmes will need to evolve with the trial, for example, in response to changes to the database, emerging issues or protocol amendments.)	No.
	2.What is the frequency with which the programme’s purpose needs to be repeated?	Need for repetition can be reduced, for example, by initial overproduction of the anticipated number of sites and schedule length. Effective allocation should be checked routinely. Frequency of these checks should be specified in monitoring plans. Increased frequency of checks earlier in the trial.
	3.Is the content fixed and predetermined?	Clearly detailed within the randomisation specification; very unlikely to change during the trial.
	4.How likely is it that programme output will need to be replicated?	Essential requirement – impact on documentation.
	5.What is the complexity of the programming and data management steps involved?	Complexity low within generation of randomisation lists but increases for dynamic allocation with a probabilistic element.
	*Validation requirements:* • *Validation is essential for both the schedule and the allocation delivery system. Extent of validation should be proportionate to the complexity of the randomisation method.* • *Use of commands in statistical software packages, or previously validated programmes, used within scope of those validations, may take the form of testing of outputs from the allocation delivery system against the randomisation specification only.*	
Empirical/probing		
	Impact area	Validity
	Impact^b^	Minor
	Likelihood^c^	Unlikely
	*Considerations:*	
	1.Do the programmes need to be flexible and fluid?	Yes. Used for exploration to understand an individual participant’s data (outliers or inconsistencies). Unlikely to be reused without modification. Likely to be affected by database changes.
	2.What is the frequency with which the programme’s purpose needs to be repeated?	Variable according to risk assessment/monitoring plan requirements.
	3.Is the content fixed and predetermined?	No. Used to develop understanding of a specific participant’s data.
	4.How likely is it that programme output will need to be replicated?	Unlikely.
	5.What is the complexity of the programming and data management steps involved?	Variable.
	*Validation requirements:* • *Validation not required*	
Complex checks: failure to identify data errors or inconsistency in the data leading to an insufficiently clean data set for analysis		
	Impact area	Safety, validity
	Impact^b^	Moderate
	Likelihood^c^	Possible
	*Considerations*:	
	1.Do the programmes need to be flexible and fluid? Is there a high risk that the programmes will need to evolve with the trial, for example, in response to changes to the database, emerging issues or protocol amendments.	No – but programme may be impacted by changes to the database.
	2.What is the frequency with which the programme’s purpose needs to be repeated?	Frequency variable and informed by data accrual and trial monitoring plan.
	3.Is the content fixed and predetermined?	Majority of complex checks identified at trial start/database development; additional checks may be identified at a later date.
	4.How likely is it that programme output will need to be replicated?	Programmes and data used in testing should be stored along with the completed test plan.
	5.What is the complexity of the programming and data management steps involved?	Used when programming complexity exceeds Clinical Data Management System (CDMS) or checks not implemented in CDMS.
	*Validation requirements:* • *Independent programming not required.* • *Test plan created using modified data set to check queries correctly identified.*	
Derived data sets: failure to include/exclude participants within analysis population; incorrect calculations, derivations or algorithms		
	Impact areas	Safety, validity
	Impact^b^	Major
	Likelihood^c^	Possible
	*Consideration*s	
	1.Do the programmes need to be flexible and fluid? Is there a high risk that the programmes will need to evolve with the trial, for example, in response to changes to the database, emerging issues or protocol amendments.	No. Impacted by changes to database. Greater chance of changes in response to deviations.
	2.What is the frequency with which the programme’s purpose needs to be repeated?	At least for each monitoring report or in conjunction with complex checks.
	3.Is the content fixed and predetermined?	Yes – may be additional requirements as trial progresses.
	4.How likely is there to be a need to replicate programme output?	Yes – need to clearly demonstrate how values obtained and membership of analysis sets.
	5.What is the complexity of the programming and data management steps involved?	Variable but highly likely to include complex programming.
	*Validation requirements:* • *Independent programming for complex derivations, calculations, algorithms and analysis populations.* • *Simple derivations or analysis population definitions by performing detailed checks of output against raw data or data listings combined with review of code/programming.*	
Statistical monitoring: failure to identify protocol deviations/violations and signals in the data		
	Impact areas	Safety, validity
	Impact^b^	Major – inclusion of safety aspects
	Likelihood^c^	Possible
	*Considerations:*	
	1.Do the programmes need to be flexible and fluid? Is there a high risk that the programmes will need to evolve with the trial, for example, in response to changes to the database, emerging issues or protocol amendments.	Yes – programmes affected by database changes. Additions may be requested.
	2.What is the frequency with which the programme’s purpose needs to be repeated?	Frequency determined within risk assessment/monitoring plan and DMC requirements.
	3.Is the content fixed and predetermined?	No – monitoring plan in place at the start of the trial. Content of DMC reports prespecified and agreed – often outlined within DMC Charter. Some unforeseen additions may be required or requested by DMC. Impacted by any changes to the database.
	4.How likely is it that programme output will need to be replicated?	Unlikely but programmes and data snapshots used should be stored.
	5.What is the complexity of the programming and data management steps involved?	Variable, dependent on trial requirements.
	*Validation requirements:* • *Statistical monitoring reports cover screening summaries to safety data summaries and primary outcome.* • *The same level of validation is not required throughout the report and should be proportionate to the purpose.* • *Independent programming required on key decision-making variables for example, dose escalation, primary outcome and variables with complex derivations and safety.* • *Check of outputs and log files maybe sufficient for other variables and summaries not considered critical, for example, key factor in decision-making.* • *No validation required for simple screening log and recruitment summaries.*	
Interim and final analysis: *(failure to ensure interpretation and future clinical decisions are based made on conclusions formed from correct analysis.)*		
	Impact areas	Safety, validity
	Impact^b^	Major/catastrophic – decisions regarding implementation or discontinuation of possibly invasive and/or costly interventions
	Likelihood^c^	Possible
	*Considerations:*	
	1.Do the programmes need to be flexible and fluid? Is there a high risk that the programmes will need to evolve with the trial, for example, in response to changes to the database, emerging issues or protocol amendments.	No – but may be impacted by database changes. Additional exploratory analyses may be requested following review of results or during peer-review process.
	2.What is the frequency with which the programme’s purpose needs to be repeated?	Low frequency. Programmes may have initial use within production of DMC reports.
	3.Is the content fixed and predetermined?	Statistical Analysis Plan with shell (or dummy) tables. Additional analyses can be requested in response to peer-review process or investigator questions.
	4.How likely is it that programme output will need to be replicated?	Replication/reconstruction from raw data may be required during regulatory inspection.
	5.What is the complexity of the programming and data management steps involved?	Variable, dependent upon trial needs.
	*Validation requirements:* • *Independent programming required on key variables, for example, primary outcome and safety.* • *Independent programming for secondary outcomes requiring more complex derivations.* • *Other secondary outcomes may be independently programmed and/or double checking of code.* • *Consideration should be given to how the outcome(s) will be used within future research or to guide implementation within clinical practice.*	

a Applied within a statistical programming framework at the second workshop in the presence of a representative from the UK regulator.

b Impact = catastrophic, major, moderate and minor.

c Likelihood = unlikely, possible, highly and probable.

## Discussion

The contribution of the statistician to the design and analysis of a clinical trial is acknowledged as essential (International Council for Harmonisation Good Clinical Practice).^1^ There is therefore a need to ensure that the statistical contribution to a trial is rigorously and transparently documented as evidenced by the ability to reconstruct the results; validation of statistical programming is a key component. A risk-proportionate approach to the management of clinical trials is supported by regulators;^12–16^ however, this is yet to be formalised throughout statistical practice.

We sought to develop and apply a risk assessment framework to support the validation of statistical programming within an academic CTU. This is benefited from the engagement of the UK CRC RCTU network, knowledge exchange between statisticians, information systems managers, quality assurance managers and the involvement of a member of the MHRA inspectorate, the UK regulator. This ensured that the approach developed was suitable across a broad range of RCTUs with diverse clinical trial portfolios and available resources. The presence of the MHRA inspector allowed a regulators perspective to be included within the discussions considering examples of previous inspection findings.

Implementing the proposed risk assessment is a non-trivial exercise requiring input from experienced statisticians. It may be argued that this increases bureaucracy and uses scarce resources itself. An alternative would be to undertake a consistent approach across all trials within an RCTU, for example, routinely validating primary outcome and safety results via independent programming. This was prevalent among many RCTUs responding to the survey and can be justified as these results are most likely to achieve impact and change clinical practice. However, this means that programmes may not be considered to be fully validated, thereby necessitating further validation for reuse. In addition, potentially greater risks are left unconsidered, for example, programming complex derivations and key secondary outcomes.

The risk assessment of the statistical programming proposed should be a component of the main risk assessment for the trial. It may need to consider the nature of the trial interventions and intentions with respect to regulatory requirements, inspections, marketing authorisation applications and use for future research. While RCTUs may wish to implement best practice across all trials in their portfolio, there may be benefits in considering these aspects given academic RCTUs frequently cite concerns regarding limited resources.^27,28^ As resources will invariably be restricted prioritisation may be considered when deciding which approach is required and the level of inherent risk. During the risk assessment and validation approach selection, this should be clearly justified.

We do not prescribe when the risk assessment for statistical programming should be undertaken. This could be beneficial when preparing a grant application, as these activities need to be costed by the RCTU, but the utility may be reduced as any risk assessment conducted at this time would be susceptible to substantial change. Changes to the risk assessment may be required due to amended specifications, error detection or accumulating knowledge across trials.

Guidance has been established on the content of statistical analysis plans for late phase trials, with an extension developed to early phase.^29,30^ Following agreement of the analysis plan, dummy tables for the clinical study report may be drafted. While there is guidance on the content of the study report, there is no best practice on simple techniques to aid reconstruction. For example, when the clinical study report is populated with results, inclusion of a line underneath each table documenting the version, location of the validated programme, its validation status, and other related validation activities could be a valuable addition. This could enable transparency and wider scrutiny of the process while also increasing understanding of the associated workload. Establishing a suite of best practices could support statisticians during reconstruction of trial results.

This article does not cover best programming guidelines. There are numerous examples available;^26^ however, they often stem from a pharmaceutical/industry setting or are specific to a particular programming language. The translation of these guidelines across a large network of academic CTUs is not straightforward due to differences in infrastructure, time and staff resources. Generating best programming guidelines for academic statisticians,^31^ independent of programming language, would be valuable future work and would support validation activities within an academic trials unit.

It is expected that statistical results can be reconstructed and that there should be consistency between any publications and the clinical study report. Reconstruction of a result may be required during a regulatory inspection and must be achievable within a reasonable timeframe. This is made increasingly difficult when statisticians are required to make changes to statistical output with tight deadlines, often as part of the peer-review process. Consideration must be given to how the validated status of the programmes can be maintained with an appropriate change control process in place. This means that the changes requested can themselves be risk assessed to determine whether further validation is required and the level of testing needed.

A clear advantage of establishing and retaining validated programmes is the reduction in the need for repeated testing. However, workshop attendees voiced concerns regarding unthinking reuse of programmes. While the primary purpose of analysing the data is to produce aggregate level information summarising the data set as a whole, the data collected on an individual trial participant tells their story during their time in the clinical trial. Achieving a quality analysis is dependent upon in-depth knowledge of the data at the participant level. This underlies the reluctance of statisticians to rerun programmes without careful exploration of data.

We have developed a framework for a risk-proportionate approach to the validation of statistical programming that can be readily adapted within academic-led CTUs. This was informed by engagement of the UK CRC RCTU network and input from the MHRA inspectorate. The proposed risk-based approach endeavours to facilitate the implementation and prioritisation of targeted quality control validation techniques while directing limited resources efficiently.

The importance of knowledge sharing and consolidating practices between information systems professionals and statisticians is apparent. Future developments should ensure this engagement continues and reflects best practice across both professions. We recommend that an adaptive risk-proportionate approach to validation is adopted where the risks associated with each statistical programme and the impacts considered are documented, mitigated and prioritised, as appropriate.
